# Indication of decompressive craniectomy in massive neonatal arterial ischemic stroke; implication from a single case

**DOI:** 10.1111/ped.70028

**Published:** 2025-05-03

**Authors:** Goro Takahashi, Joji Inamasu, Shoko Ito, Tsutomu Takahashi

**Affiliations:** ^1^ Saiseikai Utsunomiya Hospital Utsunomiya Japan; ^2^ Present address: Department of Pediatrics Keio University School of Medicine Tokyo Japan

**Keywords:** arterial ischemic stroke, brain edema, decompressive craniectomy, head circumference

In neonatal arterial ischemic stroke (NAIS), conservative management has almost always been recommended. However, the mortality rate is reportedly to be as high as 5% and the long‐term neurodevelopmental outcomes are not necessarily favorable.^1^ The term “malignant middle cerebral artery (MCA) syndrome” is adopted in childhood; decompressive craniectomy (DC), if performed before the appearance of clinical signs of herniation, can improve mortality and neurological outcomes in this syndrome.^2^ The alternative term in neonates for this syndrome is M‐NAIS (M for massive), where rapidly worsening vital signs are associated with secondary blood‐flow failure due to the original mass‐effect of NAIS; DC may also be worth consideration in M‐NAIS. We report a 3‐day‐old neonate with a M‐NAIS where an emergency DC was performed successfully. With this experience, the indication of DC for M‐NAIS is proposed.

The patient, one of the unidentical twins, was born term by elective cesarean section: 5‐min Apgar score of 10 and the birth weight of 2280 g. On the third day after birth, the patient suddenly developed apneic spells with hypoxia, that progressed within 10 minutes to respiratory arrest and was immediately intubated; the patient then became unresponsive to any stimuli. Notably, there had been no increase in the head circumference (33 cm at birth, 32 cm at diagnosis). There was no evidence of intracardiac structural abnormalities other than patent foramen ovale. There were no laboratory findings suggestive of a cause of respiratory arrest, such as hypoglycemia or electrolyte abnormalities. Hematological tests revealed no known coagulopathy for the patient and the mother; family history was also negative.

Computed tomography (CT) revealed the large hypodense legion of the right hemisphere with midline shift and multi‐focal edema of the contralateral hemisphere and thalamus (Figure 1a,b). CT angiography depicted the occlusion of the right MCA (Figure 1c). The clinical symptoms and CT findings pointed toward impending brain herniation; an emergency DC was performed immediately with parental consent.

**FIGURE 1:**
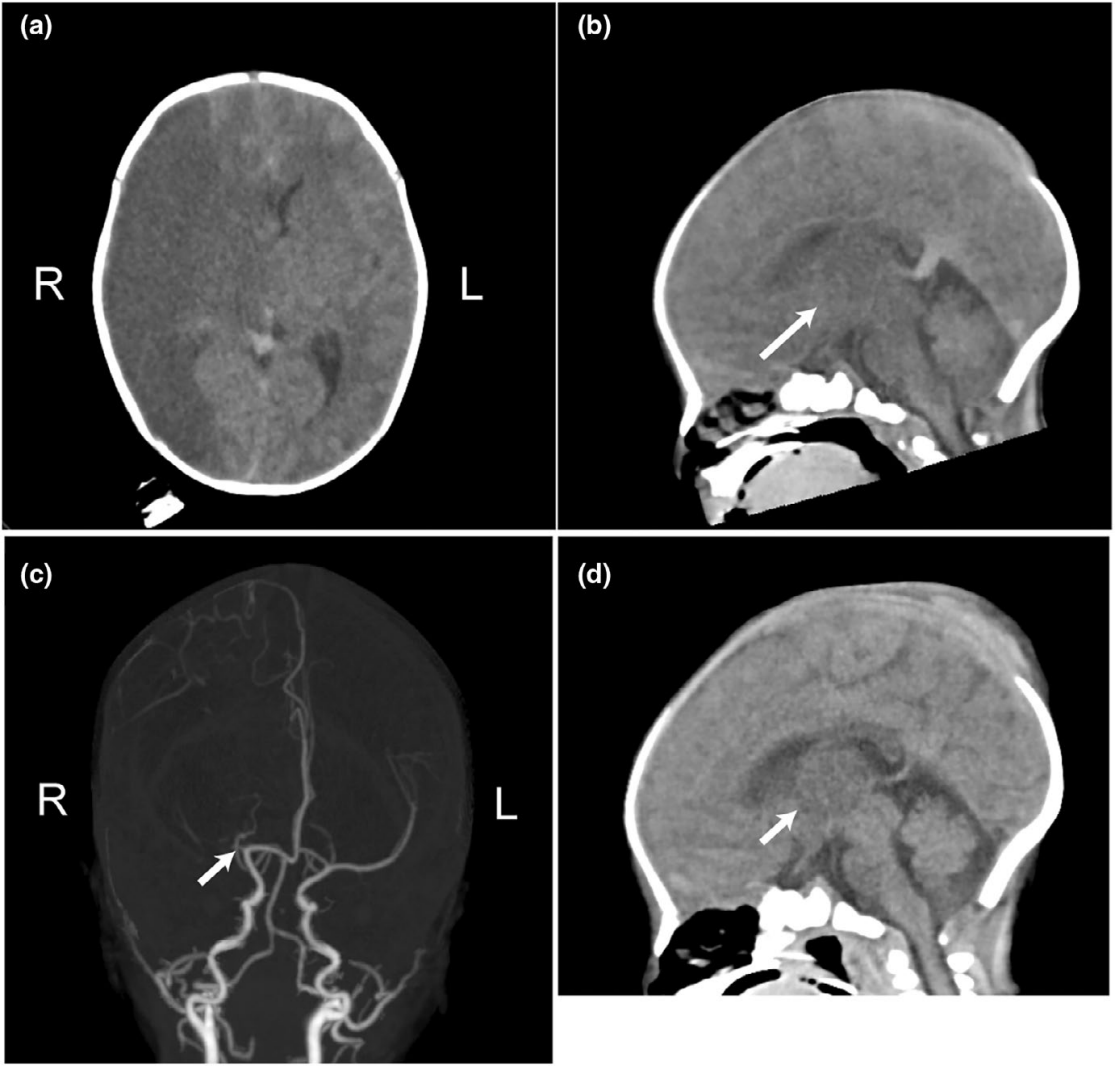
(a, b) Computed tomography (CT) at diagnosis: Diffuse homogeneous hypodensity of the right hemisphere and multi‐focal edema of the contralateral hemisphere with right to left midline shift (a) and hypodensity of the left thalamus (b, an arrow). (c) CT angiography: The right middle cerebral artery occlusion (an arrow). (d) CT at POD 1: Marked improvement of ischemia/edema of the thalamus (an arrow).

The postoperative course was uneventful. CT on the postoperative day (POD) 1 revealed remarkably improved edema of the contralateral (left) hemisphere and thalamus (Figure 1d). Edaravone and mannitol were administered preoperatively until POD 7. CT on the POD 35 showed the nearly totally regenerated skull defect; reoperation for cranioplasty was unnecessary. The patient was discharged on the POD 46 with no neurological impairment. Rehabilitation has been performed since POD 15 until now (2‐year‐old).

Developmental quotient assessed at 18 months of age was 100 (Enjoji infantile development test), equivalent to the other twin; the motor development was normal with mild clumsiness of the left hand. The head circumference was within the normal range at 2‐year‐old.

Among guidelines for management of childhood‐stroke, only American Heart Association lists the DC as a treatment option for neonate when a massive mass effect is present; otherwise, no specific indication criteria are mentioned.^3^

Ischemia/edema of the left thalamus in the present case is suggestive of decreased posterior circulation. When NAIS is associated with the impaired posterior circulation in addition to the main infarcted region, it may better be taken as a sign of massive mass effect progressing beyond the tentorial sinus.^4^ Additionally, in this case, multiple low‐density areas of the contralateral hemisphere suggested impaired venous circulation.

In our case, no acute enlargement of the head circumference was observed. The open cranial sutures of neonates compensates elevation of intracranial pressure (ICP). However, an animal study showed that the ICP increase was not buffered by cranial suture dissection in case of rapid increase.^5^ Additionally, there was generally no difference in head circumference between M‐NAIS and non‐M‐NAIS.^4^ With those findings taken together with the case presented here, judgment regarding the severity of ICP elevation should never be solely dependent upon the absence or presence of the head enlargement.

To the best of our knowledge, there have been no reports on the use of DC for M‐NAIS. Its safety and efficacy remain to be validated by future studies with larger patient population and longer follow‐up period.

In conclusion, emergency DC may be considered as a treatment option for M‐NAIS, when there are rapidly worsening vital signs and evidence of impaired multiple‐system circulations.

## AUTHOR CONTRIBUTIONS

G.T., J.I., S.I., and T.T. cared for the patient and wrote this paper. All the authors read and approved the final manuscript.

## CONFLICT OF INTEREST STATEMENT

The authors declare no conflict of interest.

## ETHICS STATEMENT

Written informed consent was obtained from the patient's parents for the publication of this report.
